# Hypohydration impairs endurance performance: a blinded study

**DOI:** 10.14814/phy2.13315

**Published:** 2017-06-22

**Authors:** Lewis J. James, Jodie Moss, Joshua Henry, Charikleia Papadopoulou, Stephen A. Mears

**Affiliations:** ^1^ School of Sport, Exercise and Health SciencesLoughborough UniversityLeicestershireLE11 3TUUnited Kingdom

**Keywords:** Dehydration, drink, hydration, placebo effect, time trial, water balance

## Abstract

The general scientific consensus is that starting exercise with hypohydration >2% body mass impairs endurance performance/capacity, but most previous studies might be confounded by a lack of subject blinding. This study examined the effect of hypohydration in a single blind manner using combined oral and intragastric rehydration to manipulate hydration status. After familiarization, seven active males (mean ± SD: age 25 ± 2 years, height 1.79 ± 0.07, body mass 78.6 ± 6.2, *V*O_2peak_ 48 ± 7 mL·kg·min^−1^) completed two randomized trials at 34°C. Trials involved an intermittent exercise preload (8 × 15 min exercise/5 min rest), followed by a 15‐min all‐out performance test on a cycle ergometer. During the preload, water was ingested orally every 10 min (0.2 mL·kg body mass^−1^). Additional water was infused into the stomach via a gastric feeding tube to replace sweat loss (EU) or induce hypohydration of ~2.5% body mass (HYP). Blood samples were drawn and thirst sensation rated before, during, and after exercise. Body mass loss during the preload was greater (2.4 ± 0.2% vs. 0.1 ± 0.1%; *P < *0.001), while work completed during the performance test was lower (152 ± 24 kJ vs. 165 ± 22 kJ; *P < *0.05) during HYP. At the end of the preload, heart rate, RPE, serum osmolality, and thirst were greater and plasma volume lower during HYP (*P < *0.05). These results provide novel data demonstrating that exercise performance in the heat is impaired by hypohydration, even when subjects are blinded to the intervention.

## Introduction

Endurance exercise increases metabolic heat production and consequently sweat rate is increased to facilitate heat loss through evaporative cooling. Water intake during endurance exercise is rarely sufficient to replace water lost, meaning hypohydration is common at the end of prolonged exercise (Sawka et al. 2007). Most studies have reported that starting endurance exercise hypohydrated reduces performance/capacity (Armstrong et al. 1985; Walsh et al. 1994; Below et al. 1995; McConell et al. 1997; Cheuvront et al. 2005; Ebert et al. 2007; Stearns et al. 2009; Castellani et al. 2010; Kenefick et al. 2010; Bardis et al. 2013a,b; Davis et al. 2014; Fleming and James 2014; Logan‐Sprenger et al. 2015), with a few exceptions that have reported no difference in performance between euhydrated and hypohydrated trials (McConell et al. 1999; Stewart et al. 2014; Cheung et al. 2015; Wall et al. 2015; Berkulo et al. 2016). In contrast, the results of studies where subjects start an exercise test euhydrated and hypohydration develops during exercise have yielded less consistent results (Goulet 2011).

Hypohydration results in a reduction in plasma volume (i.e., hypovolemia) and leads to a cascade of effects that increase cardiovascular strain (Sawka et al. 2015), possibly limiting maximal oxygen uptake (Cheuvront and Kenefick 2014). These effects are further exacerbated when endurance exercise is undertaken in a hot environment (Sawka et al. 2015). While there is a clear mechanistic basis for the reported reduction in endurance with hypohydration, to date the majority of studies are potentially limited by their methodology (Cotter et al. 2014). The overtness of the methods used to induce hypohydration (i.e., fluid restriction with or without exercise, heat exposure, diuretic administration, etc.) has meant that subjects are generally aware which trial they are undertaking (hypohydrated or euhydrated). Therefore, it cannot be discounted that the subject's expectation of how hypohydration impacts endurance performance might, at least partially, explain the results of previous studies (McClung and Collins 2007).

Notable among the studies that have reported similar exercise performance between euhydrated and hypohydrated trials are two recent studies (Cheung et al. 2015; Wall et al. 2015) that both used intravenous rehydration to blind subjects to the manipulation of their hydration status (0% vs. 2–3% hypohydration). These findings are of great interest and importance as they clearly suggest that a lack of study blinding might have confounded previous studies. However, the methods employed might somewhat limit interpretation of these data. Hypohydration induced by exercise generally results in decreased plasma volume and increased serum osmolality (i.e., hypertonic hypovolemia), as well as increased thirst sensation (Cheuvront and Kenefick 2014). These variables represent the main physiological and perceptual responses to hypohydration. Oral rehydration during exercise attenuates these perturbations (Dugas et al. 2009; Cheung et al. 2015; Wall et al. 2015). In contrast, infusion of approximately isotonic saline to replace sweat losses, as used by Wall et al. (2015) and Cheung et al. (2015), means the increase in serum osmolality remains irrespective of hydration status. Furthermore, neither study allowed any oral fluid ingestion, which might be important for thirst perception (Figaro and Mack 1997) and exercise performance (Arnaoutis et al. 2012), with oropharyngeal responses possibly playing an important role in the rehydration process (Casa et al. 2008).

Additional studies that not only blind subjects to alterations in hydration status, but also produce typical physiological and perceptual responses associated with hypohydration (i.e., decreased plasma volume, as well as increased serum osmolality and thirst) are warranted. Therefore, this study used combined intragastric and oral rehydration to examine the impact of hydration status on endurance performance in the heat with subjects unaware that their hydration status was being manipulated. It was hypothesized that hydration status would not influence endurance performance.

## Materials and Methods

### Subjects

After approval by the university's Ethics Committee, seven healthy men (mean [±SD]: age 25 ± 2 years, height 1.79 ± 0.07, body mass 78.6 ± 6.2, *V*O_2peak_ 48 ± 7 mL·kg^−1^·min^−1^) completed the study. Subjects completed a medical screening questionnaire and provided written consent before participation. All subjects were physically active and had previously volunteered for experiments involving stationary cycling, but none were trained cyclists or heat acclimated at the time of the study. Each subject completed four preliminary trials, followed by two experimental trials. Eight subjects were originally recruited for the study, but one subject dropped out after the third preliminary trial due to the time commitment required to complete the study. Using the data of Kenefick et al. (2010), an *α* of 0.05 and a statistical power of 0.8, it was estimated that six subjects would be required to reject the null hypothesis for the primary outcome (i.e., endurance performance).

### Preliminary trials

During the first preliminary trial, height and body mass were recorded, before cycling *V*O_2peak_ and peak power output (PPO) (Lode Corival, Groningen, the Netherlands) were determined. Exercise began at 95 W and increased by 35 W every 3 min until volitional exhaustion. Heart rate, rating of perceived exertion (RPE) (Borg 1982), and 1 min expired gas samples were collected at the end of each increment and at exhaustion. During the second and third preliminary trials, subjects completed a 5‐min warm up at 50% PPO, followed by the 15 min performance test used in experimental trials. For some subjects, the second preliminary trial took place on the same day as the *V*O_2peak_ test. During the fourth preliminary trial, subjects were familiarized with the entire experimental protocol (i.e., the preload followed by the exercise performance test).

### Pretrial standardization

All food consumed in the 24 h before the first experimental trial and any habitual physical activity undertaken in the 48 h before the first experimental trial was recorded by subjects and replicated before the second experimental trial. During this time, subjects refrained from strenuous exercise or alcohol ingestion. To help ensure adequate pre‐exercise nutritional intake, subjects were provided with a standardized evening meal (providing 3.75 g·kg body mass^−1^ of carbohydrate) to consume between 7 and 10 pm the evening before each trial and breakfast (providing 1 g·kg body mass^−1^ of carbohydrate) to consume 2 h before each trial. To ensure adequate fluid intake prior to experimental trials, subjects were provided fluid at 40 mL·kg body mass^−1^ to consume the day before trials. This was distributed as 8 mL·kg body mass^−1^ water during the morning, 16 mL·kg body mass^−1^ water during the afternoon, and 16 mL·kg body mass^−1^ of sports drink during the evening. In the morning of the trial, subjects consumed 8 mL·kg body mass^−1^ of sports drink as part of the standardized breakfast. For the water provided in the morning and afternoon on the day before trials, subjects were able to substitute other fluids for a portion or all of this water before their first trial, as long as the fluid intake was matched before their second trial. In these cases, these changes in fluid selection were recorded in the food diary and replicated for the second trial. Subjects ingested a disposable temperature sensor capsule (CorTemp sensor, HQInc, Palmetto) at 10 pm the night before each trial for measurement of T_GI_ during trials.

### Experimental trials

Trials began in the morning at a time standardized for each subject (8–9 am) and were separated by ≥7 days. Upon arrival, nude body mass was measured and subjects rated their thirst, using a 100‐mm visual analog scale: “How thirsty do you feel now?,” with anchors of “not at all” and “extremely” placed at 0 mm and 100 mm, respectively. A 20 g plastic cannula was inserted into an antecubital vein, before subjects orally inserted an 8 Fr gastric feeding tube (Sonde Gastro‐duodenal Type Levin, Vygon Ltd., Cirencester, UK) to the base of their stomach. The length of tube inserted was estimated based on the subject's height and was typically 50–60 cm. Subjects then attached a heart rate monitor (Polar Beat, Kempele, Finland). After 15 min seated rest in a neutral environment (21.6 ± 0.9°C, 50 ± 6% relative humidity), a blood sample was drawn, heart rate and T_GI_ were recorded, and thermal sensation (Sawka et al. 1985) was rated (pre‐exercise).

Subjects then entered a controlled environment (34°C and 50% relative humidity) and completed an exercise preload consisting of eight blocks of 15 min cycling at 50% PPO, each separated by 5 min rest in the chamber. No specific facing air flow was provided, but air circulation within the environmental chamber provides a wind speed of ~0.3–0.4 m·sec^−1^. Heart rate, T_GI_, RPE, and thermal sensation were measured during the last min of each 15 min exercise block. Stomach fullness and bloating were also measured on 12‐point scales, with 0, 4, 8, and 12 representing “neutral,” “slightly,” “very,” and “extremely,” respectively. Expired gas samples were collected during the final minute of the fourth (74–75 min) and eighth (154–155 min) exercise blocks. Additional blood samples were drawn immediately after the first (15 min), fourth (75 min), and eighth (155 min) blocks of exercise with subjects on the cycle ergometer. The gastric feeding tube was then removed, nude body mass was measured, and subjects again rated their thirst during a further 5 min rest. Subjects then completed a 15‐min cycling performance test, after which a final blood sample was taken with subjects still on the cycle ergometer (post‐PT), and after a short recovery period thirst was rated and a final nude body mass measurement was made.

### Performance test

For the performance test, workload was initially set to 90% PPO and subjects could increase or decrease the workload by pressing up or down on the ergometer's console. Subjects were instructed to complete as much work as possible in the 15 min. They received no feedback related to work rate, work completed (kJ), revolution·min^−1^, heart rate, or T_GI_, but could see a clock displaying time remaining. Standard instructions were given to subjects before each performance test and no encouragement was provided. Every 5 min, work completed, heart rate, T_GI_, and environmental conditions were recorded without disturbing the subject. Unpublished data from our laboratory using 12 similarly trained males (i.e., VO_2peak_ 51 ± 7 mL·kg^−1^·min^−1^), performing four 15 min performance tests, showed a mean coefficient of variation (CV) of 1.0% (range: 0.2–1.8%) after two familiarization trials.

### Manipulation of hydration status and study blinding

During both trials, subject's orally ingested 0.2 mL·kg body mass^−1^ water every 10 min of the preload (total of 260 ± 50 mL). Additional water was infused directly into the stomach through the gastric feeding tube every 5 min during the preload. The volume of infused water was manipulated to either replace sweat lost and maintain euhydration (EU; total of 1956 ± 209 mL) or produce hypohydration of ~2.5% body mass at the end of the preload (HYP; total of 123 ± 56 mL). The infusion process was identical in each trial, with an investigator connecting a syringe to the gastric feeding tube and delivering the water over ~1 min. During the hypohydrated trial, the investigator performed a dummy infusion lasting the same length of time. The subjects could not feel or hear the infusion occurring. During the fourth preliminary trial, the volume of fluid infused into the stomach was estimated to maintain euhydration, as sweat rate for the preload exercise was unknown.

Subjects were told that the study was investigating drinks of different composition and that the gastric feeding tube was used as they would have been able to identify the drinks based on their flavor profiles. The gastric feeding tube was taped behind the ear and onto the upper back, so the drink was infused outside the subject's field of vision. The infused water was maintained at ~36°C to remove any sensation of cold water passing through the tube. All subjects were interviewed upon completion of the study to determine the success of the blinding. Subjects were initially asked if they thought they knew what the difference between the two drinks was, and to identify any other differences between the two trials. They were then informed that manipulation of drink composition was not the real aim of the study and asked if they could guess the real aim. Finally, they were told the study aim and asked if they could identify the hypohydrated and euhydrated trials.

### Analytical methods

For each blood sample, 5 mL blood was dispensed into a tube containing a clotting catalyst (Sarstedt AG & Co., Nümbrecht, Germany) and 2.5 mL was mixed with EDTA (1.75 mg·mL^−1^; Sarstedt AG & Co., Nümbrecht, Germany), before serum and plasma were separated by centrifugation (1700*g*, 10 min, 4°C). Serum was refrigerated before analysis for osmolality by freezing point depression (Osmomat 030 Cryoscopic Osmometer; Gonotec, Berlin, Germany). Plasma was frozen at −20°C, before analysis for arginine vasopressin concentration by ELISA (Enzo Life Sciences, Exeter, UK; intra‐assay CV of 9.0%). The remaining 2.5 mL blood was mixed with EDTA and used for the determination of hemoglobin concentration (cyanmethemoglobin method) and hematocrit (microcentrifugation), before calculation of change in plasma volume relative to 0 min (Dill and Costill 1974). All analyses were performed on all samples, with the exception of arginine vasopressin concentration, which was not determined in the 75 min sample due to funding constraints. Expired gas was collected into a Douglas bag, and analyzed for O_2_ and CO_2_ concentration (1400 series, Servomex, East Sussex, UK), volume (Harvard Dry Gas Meter, Harvard Ltd., Kent, UK) and temperature (Edale Thermistor, Cambridge, UK), before *V*O_2_ and respiratory exchange ratio, as well as carbohydrate and fat oxidation were determined (Frayn 1983). Sweat loss was determined from the change in body mass during exercise after subtracting the weight of any urine produced, and was determined for both the preload and the performance test. It was assumed that 1 kg body mass = 1 L sweat.

### Statistical analysis

Data were analyzed using SPSS 20 (V) (Chicago, IL). All data were checked for normality using a Shapiro–Wilk test. Data containing two factors were then analyzed using two‐way repeated measures ANOVA. When the Mauchly's test indicated that the assumption of sphericity had been violated, the degrees of freedom were corrected using the Greenhouse‐Geisser estimate. Significant differences were located using paired *t* tests for normally distributed data or Wilcoxon signed‐rank tests for non‐normally distributed data. The Holm–Bonferroni correction for multiple comparisons was used to control the family‐wise error rate. Data containing one factor were analyzed using paired *t* tests or Wilcoxon signed‐rank tests, as appropriate. Differences between datasets were considered significant when *P *≤* *0.05. Data are presented as mean ± SD.

## Results

### Pretrial measures

Pre‐exercise body mass (*P = *0.703), serum osmolality (*P = *0.878), plasma arginine vasopressin concentration (*P = *0.856), and thirst (*P = *0.413) were not different between trials (Table 1), indicating subjects started each trial in a similar hydration state.

**Table 1:** Body mass (kg), change in body mass relative to 0 min (%), change in plasma volume relative to 0 min (%), serum osmolality (mosmol kg^−1^), plasma arginine vasopressin (pg mL^−1^), and thirst (0‐100 mm) during a 155‐min intermittent cycling preload (8 × 15 min exercise separated by 5 min rest), followed by a 15‐min performance test in a hot environment during the hypohydrated (HYP) and euhydrated (EU) trials

	0 min	15 min	75 min	155 min	Post‐PT
Body mass (kg)					
EU	78.2 ± 7.3	—	—	78.1 ± 7.2	77.6 ± 7.2^a^
HYP	77.9 ± 6.7	—	—	76.1 ± 6.5^a^^,^ b	75.6 ± 6.5^a^^,^ b
Change in body mass (%)					
EU	0 ± 0	—	—	−0.1 ± 0.1	−0.7 ± 0.1^a^
HYP	0 ± 0	—	—	−2.4 ± 0.2^a^^,^ b	−3.0 ± 0.3^a^^,^ b
Change in plasma volume (%)					
EU	0 ± 0	−6.1 ± 2.2^a^	−6.8 ± 2.2^a^	−7.2 ± 2.9^a^	−11.9 ± 3.1^a^
HYP	0 ± 0	−6.4 ± 2.0^a^	−9.4 ± 2.7^a^^,^ b	−12.3 ± 2.3^a^^,^ b	−15.0 ± 2.4^a^^,^ b
Serum osmolality (mosmol kg^−1^)					
EU	284 ± 2	288 ± 2^a^	286 ± 4	285 ± 3	290 ± 2^a^
HYP	284 ± 3	288 ± 3^a^	291 ± 3^a^	294 ± 3^a^^,^ b	299 ± 3^a^^,^ b
Arginine vasopressin (pg mL^−1^)					
EU	2.25 ± 0.78	1.99 ± 0.31	—	1.83 ± 0.25	2.36 ± 0.67
HYP	2.17 ± 0.31	2.30 ± 0.47	—	4.12 ± 1.96^a^^,^ b	8.84 ± 22^a^^,^ b
Thirst (mm)					
EU	37 ± 22	—	—	45 ± 18	65 ± 15
HYP	30 ± 7	—	—	73 ± 20^a^^,^ b	85 ± 8^a^^,^ b

Values are mean (SD).

a Significantly different from pre‐exercise.

b Significantly different from EU.

### Fluid balance measures

There was an interaction effect for body mass (Table 1), with body mass reduced during the preload in HYP (*P < *0.001), but not EU (*P = *0.094). After the performance test, body mass was reduced during both HYP (*P < *0.001) and EU (*P < *0.05), but to a greater degree during HYP (*P < *0.001). Sweat loss was not different between trials during either the preload (EU 2.3 ± 0.4 kg, HYP 2.3 ± 0.4 kg; *P *=* *0.164) or performance test (EU 0.4 ± 0.0 kg, HYP 0.4 ± 0.1 kg; *P *=* *0.798).

There were interaction effects for the change in plasma volume (*P < *0.001), serum osmolality (*P < *0.001), plasma arginine vasopressin concentration (*P < *0.001), and thirst (*P < *0.001). Plasma volume (Table 1) decreased by ~6% from pre‐exercise to 15 min in both trials (*P < *0.001) and remained decreased relative to pre‐exercise at all subsequent time points on both trials (*P < *0.001). The reduction in plasma volume was greater during HYP than EU at 75 min (*P < *0.05), 155 min (*P < *0.05), and post‐PT (*P < *0.05). Serum osmolality (Table 1) increased by ~4 mosmol·kg^−1^ in both trials (*P < *0.05) from pre‐exercise to 15 min, remaining increased at all subsequent time points during HYP (*P < *0.01), but only post‐PT during EU (*P < *0.05). Serum osmolality was greater during HYP than EU at 155 min (*P < *0.001) and post‐PT (*P < *0.01). Plasma arginine vasopressin concentration (Table 1) was increased at 155 min and post‐PT during HYP (*P < *0.05), and was greater at 155 min and post‐PT during HYP compared to EU (*P < *0.05). Compared to pre‐exercise, thirst (Table 1) was increased at 155 min and post‐PT during HYP (*P < *0.01). Additionally, thirst was greater at 155 min (*P < *0.05) and post‐PT (*P < *0.01) during HYP compared to EU. Between the preload and performance test, two subjects produced urine in both trials, while one subject produced urine on the EU trial only.

### Preload exercise responses

**Figure 1:**
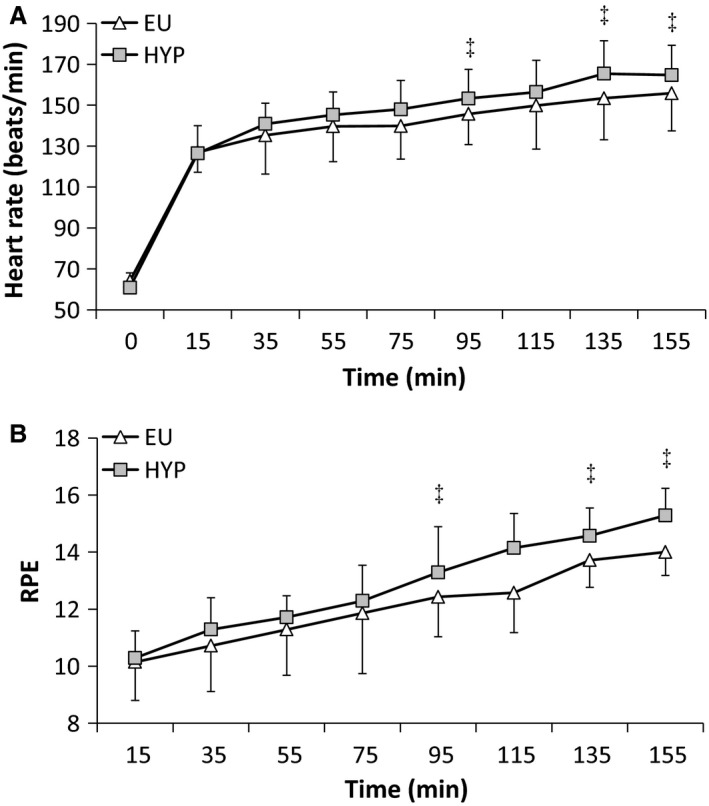
(A) Heart rate (beat min^−1^) and (B) rating of perceived exertion (RPE) responses during a 155‐min intermittent cycling preload (8 × 15 min exercise separated by 5 min rest in a hot environment) during the hypohydrated (HYP) and euhydrated (EU) trials. Values are mean ± SD. ‡Significantly different from EU.

**Figure 2:**
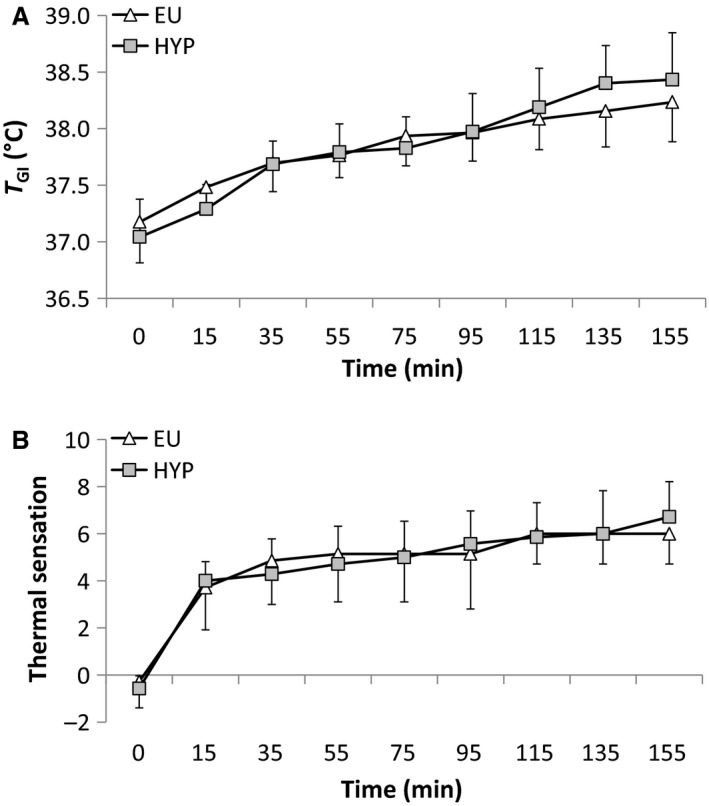
(A) Gastrointestinal temperature (°C) and (B) thermal sensation responses during a 155‐min intermittent cycling preload (8 × 15 min exercise separated by 5 min rest in a hot environment) during the hypohydrated (HYP) and euhydrated (EU) trials. Values are mean ± SD. ‡Significantly different from EU.

**Figure 3:**
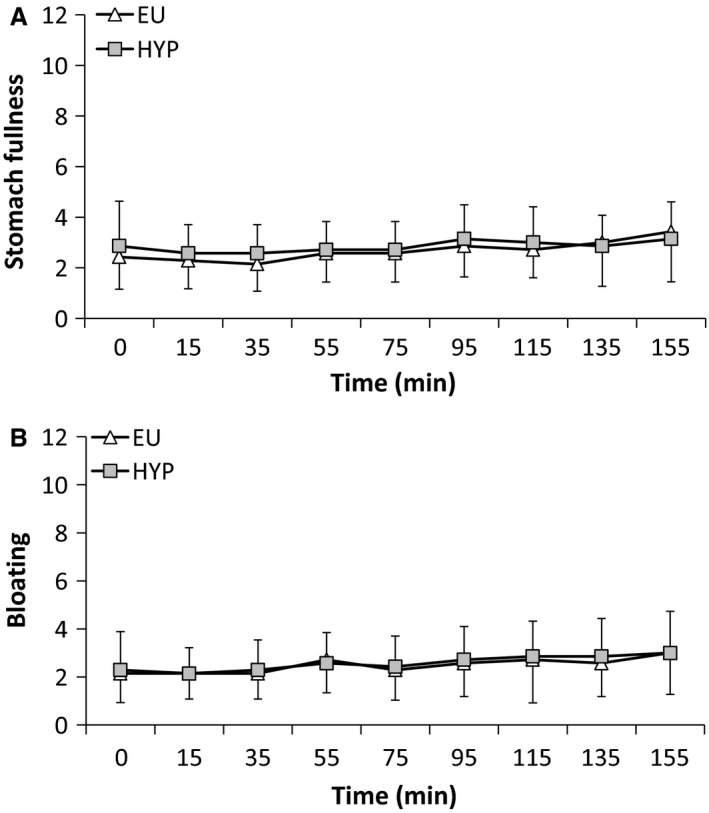
(A) Stomach fullness and (B) bloating during a 155‐min intermittent cycling preload (8 × 15 min exercise separated by 5 min rest in a hot environment) during the hypohydrated (HYP) and euhydrated (EU) trials. Values are mean ± SD.

There were main effects of time for heart rate, RPE, T_GI_, and thermal sensation (all *P < *0.001), with all increasing progressively throughout the preload. During the preload, there was an interaction effect for heart rate (Fig. 1A; *P < *0.01), which increased to a greater extent during HYP (*P < *0.001). Heart rate was greater during HYP than EU at 95, 135, and 155 min (*P < *0.05). There was a main effect of trial (*P < *0.01), but no interaction effect (*P = *0.160) for RPE (Fig. 1B). RPE was greater during HYP than EU at 95, 135, and 155 min. There was no main effect of trial (*P = *0.874) or interaction effect (*P = *0.111) for T_GI_ (Fig. 2A). Similarly, there was no main effect of trial (*P = *0.969) or interaction effect (*P = *0.525) for thermal sensation (Fig. 2B). There were no time (*P = *0.565, *P = *0.731), trial (*P = *0.524, *P = *0.196), or interaction effects (*P = *0.869, *P = *0.990) for stomach fullness (Fig. 3A) or bloating (Fig. 3B). There were time (all *P < *0.001), but no trial (*P *≥* *0.625) or interaction (*P *≥* *0.193) effects for VO_2_, RER, and substrate utilization (data not shown). *V*O_2_ and fat oxidation increased, while RER and carbohydrate oxidation decreased between 75 min and 155 min.

### Performance test

**Figure 4:**
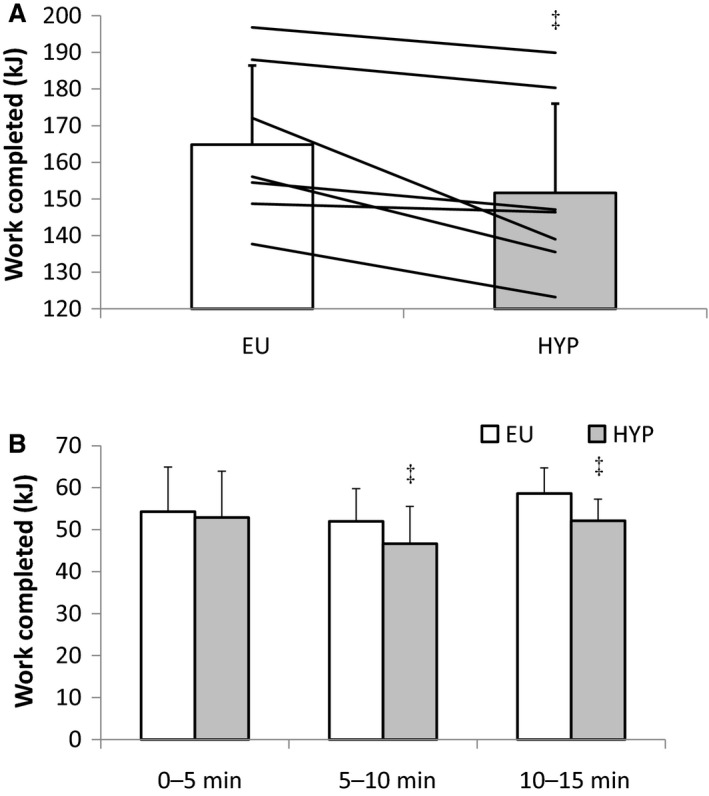
(A) Work completed in the 15 min performance test (kJ) and (B) work completed during each 5 min of the performance test in a hot environment during the hypohydrated (HYP) and euhydrated (EU) trials. Bars are mean ± SD. Lines represent individual values. ‡Significantly different from EU.

Total work completed during the performance test was 8.1 ± 6.4% greater during EU than HYP (Fig. 4A). When the performance test was separated into 5 min blocks, a greater amount of work was completed during EU compared to HYP between 5 and 10 min (*P < *0.05) and 10 and 15 min (*P < *0.05), but not between 0 and 5 min (*P = *0.211) (Fig. 4B). Heart rate (*P = *0.942) and T_GI_ (*P = *0.103) responses during the performance test were similar between trials. At the end of the performance test, heart rate was 184 ± 14 beat min^−1^ and 182 ± 11 beat min^−1^, while T_GI_ was 38.32 ± 0.53°C and 38.67 ± 0.47°C during EU and HYP, respectively.

### Posttrial interview

No subject indicated that they thought hydration status had been manipulated in the first question of the posttrial interview. When subjects were told that they had been deceived and that the study was not investigating drink composition, only one subject correctly guessed that manipulation of hydration status was the true aim. Once subjects were told their hydration status had been manipulated during the trials, all subjects correctly identified the trial order.

## Discussion

The aim of this study was to investigate the effect of hypohydration on endurance performance with subjects blinded to the intervention and therefore unaware that their hydration status was being manipulated. In contrast to our hypothesis and the findings of Wall et al. (2015) and Cheung et al. (2015), starting the performance test with hypohydration equivalent to ~2.4% body mass decreased endurance performance by ~8% compared to the euhydrated trial.

Most previous work has reported decreased endurance performance/capacity when exercise is started in a hypohydrated compared to euhydrated state (Armstrong et al. 1985; Walsh et al. 1994; Below et al. 1995; McConell et al. 1997; Cheuvront et al. 2005; Ebert et al. 2007; Stearns et al. 2009; Castellani et al. 2010; Kenefick et al. 2010; Bardis et al. 2013a,b; Davis et al. 2014; Fleming and James 2014; Logan‐Sprenger et al. 2015), with a few exceptions where hydration status did not influence performance (McConell et al. 1999; Stewart et al. 2014; Cheung et al. 2015; Wall et al. 2015; Berkulo et al. 2016). The results of the present study extend those of previous studies and for the first time demonstrate that even when subjects are blinded to the manipulation of their hydration status, hypohydration impairs endurance performance, at least in lesser trained, nonheat acclimated males. The present findings do not discount the existence of a nocebo effect associated with hypohydration (or fluid restriction), but they do demonstrate that hypohydration of ~2.4% body mass results in a measurable decrement in performance.

Hypohydration appears to impair endurance performance through a combination of physiological and perceptual factors seemingly driven by hypovolemia (Sawka et al. 2015). This hypovolemia, and accompanying serum hyperosmolality, cause a cascade of effects that likely act in combination to limit endurance performance. These might include reduced muscle (Gonzalez‐Alonso et al. 1998) and cerebral (Trangmar et al. 2014) blood flow, increased cardiovascular strain (Montain and Coyle 1999), impaired thermoregulation and increased core temperature (Sawka et al. 1985), increased perceived exertion (Walsh et al. 1994; Casa et al. 2010, Castellani et al. 2010; Kenefick et al. 2010; Fleming and James 2014; Logan‐Sprenger et al. 2015), and increased thirst (Dugas et al. 2009; Casa et al. 2010). The present study was successful in producing physiological and perceptual responses consistent with hypohydration and euhydration. Plasma volume was decreased, while heart rate, RPE, serum osmolality, arginine vasopressin, and thirst were all increased in the hypohydrated trial. While there was no difference in gastrointestinal temperature between trials, mean values were greater in the hypohydrated trials and it may be that the study was simply underpowered to detect differences in body temperature. While two previous studies have examined the impact of hypohydration in a blinded manner (Cheung et al. 2015; Wall et al. 2015), neither of these studies successfully induced all physiological and perceptual responses consistent with hypohydration.

It seems likely that some combination of the methods used and the subject populations studied might account for the different results observed in the present study compared to these two previous blinded hydration studies (Cheung et al. 2015; Wall et al. 2015). The infusion of approximately isotonic rehydration fluids in previous blinded hydration studies meant that the serum hyperosmolality produced by dehydration during exercise was also present in the rehydrated trials. In contrast, the use of water (i.e., hypotonic fluid) for rehydration in the present study prevented serum hyperosmolality, as reported in previous studies (Kenefick et al. 2010; Bardis et al. 2013a,b; Logan‐Sprenger et al. 2015). Given the role serum osmolality plays in regulating physiological and behavioral responses to alterations in fluid balance (Cheuvront and Kenefick 2014), it seems likely that appropriate manipulation of serum osmolality response might be an important methodological consideration for a blinded hydration study. Additionally, in both previous blinded hydration studies, oral rehydration (i.e., the swallowing of fluid) was completely restricted. Wall et al. (2015) reported no difference in thirst between trials, despite differences in hydration of up to 3% body mass. In contrast, Cheung et al. (2015) permitted or restricted mouth rinsing of water during trials to produce hypohydrated and euhydrated trials with subjects either thirsty or not thirsty, and neither thirst nor hydration status impacted exercise performance. Arnaoutis et al. (2012) demonstrated that in a hypohydrated state, swallowing (25 mL·5 min^−1^), but not rinsing and expectorating (25 mL·5 min^−1^) water, enhanced performance. Thus, it would appear that manipulating thirst sensation by mouth rinsing water might act to alleviate symptoms of dry mouth, but not physiological thirst and dipsogenic drive, as suggested by Cheung et al. (2015). In the present study, a small volume of water was ingested orally (i.e., swallowed) in both trials (260 ± 50 mL), with the remaining water in each trial infused directly into the stomach. This combination of water delivery techniques maintained thirst at pre‐exercise levels in the euhydrated trial and increased thirst in the hypohydrated trial.

The training status of the subjects used in the present and previous blinded hydration studies (Cheung et al. 2015; Wall et al. 2015) also represents a possible difference that might account for the divergent results. While the subjects in the present study were familiar with cycling and all had previously taken part in experiments involving laboratory‐based cycling exercise, none was a trained cyclist. In contrast, Wall et al. (2015) and Cheung et al. (2015) both used trained cyclists. Merry et al. (2010) observed that training status modulates the effect of hypohydration on cardiovascular/thermoregulatory function, but not endurance performance. Similarly, other previous studies have reported performance impairments with hypohydration whether subjects are endurance trained (Armstrong et al. 1985; Walsh et al. 1994; McConell et al. 1997; Ebert et al. 2007; Stearns et al. 2009; Bardis et al. 2013a,b; Logan‐Sprenger et al. 2015) or untrained (Cheuvront et al. 2005; Castellani et al. 2010; Kenefick et al. 2010; Merry et al. 2010; Fleming and James 2014), suggesting that irrespective of training status, hypohydration might impair endurance performance. Fleming and James (2014) demonstrated that familiarity with the methods used to induce hypohydration attenuates the performance impairment caused by hypohydration. It may be that familiarity with the hypohydration stimulus and not training status attenuates the deleterious effects of hypohydration. While the subjects of Wall et al. (2015) and Cheung et al. (2015) were not specifically heat acclimated, the studies were conducted at times when the subjects were likely doing at least some of their training in a warm environment. Thus, they may have experienced fluid restriction combined with cycling exercise in the heat during training, and this experience might have attenuated the impact of hypohydration on performance. Clearly further work is needed to confirm this hypothesis.

The results of the present study might only apply to situations where facing air flow is low (e.g., indoor training sessions). Outdoor cycling is accompanied by facing air flow similar to cycling speed, unless the cyclist is drafting. In contrast, stationary cycling, used in indoor training sessions, generally takes place in near wind still conditions, which alters thermoregulation during exercise (Saunders et al. 2005). The low facing airflow used in previous studies has been postulated to exacerbate the hypohydration‐induced performance impairment (Saunders et al. 2005; Wall et al. 2015). With the exception of one study which used a high speed fan during indoor cycling (Wall et al. 2015), the majority of previous cycling studies (Walsh et al. 1994; Below et al. 1995; McConell et al. 1997,1999; Ebert et al. 2007; Kenefick et al. 2010; Logan‐Sprenger et al. 2015; Cheuvront et al. 2005; Castellani et al. 2010; Stewart et al. 2014) examining changes in hydration in excess of 2% body mass have used facing air flow that is well below estimated cycling speed (i.e., 0–3.2 m sec^−1^ vs. >8–10 m sec^−1^). Interestingly, there was no significant difference between trials for T_GI_ or thermal sensation, suggesting the observed effects might not have been mediated by changes in thermoregulation. The decision to not provide facing air flow in the present study was taken so that convective cooling was similar to that used in the majority of previous studies reporting hypohydration to impair performance (Walsh et al. 1994; Castellani et al. 2010; Cheuvront et al. 2005; Fleming and James 2014; Kenefick et al. 2010; Logan‐Sprenger et al. 2015; Bardis et al. 2013a). This allowed the impact of study blinding to be investigated, but whether the results of the present study would extend to “real‐world” outdoor exercise performance where facing air flow is greater is not known at this time. This should be the focus of future studies to address this gap in our current knowledge.

## Conclusions

In conclusion, the present study successfully manipulated key physiological and perceptual responses in a manner consistent with hypohydration, while also blinding subjects to the intervention. Therefore, this study demonstrates, for the first time using a blinded study design, that starting exercise hypohydrated (~2.4% body mass) impairs cycling performance in the heat, at least in a population of active, but not specifically cycling trained, nonheat acclimated males.

## Conflicts of Interest

L. J. J. has previously received funding for his research from PepsiCo, Inc. and has previously performed consultancy work for Lucozade Ribena Suntory. This previous funding/consultancy activities are in no way linked to the present study and have always been paid directly to L. J. J.'s employer, not L. J. J.

## References

[phy213315-bib-0001] Armstrong, L. E. , D. L. Costill , and W. J. Fink . 1985 Influence of diuretic‐induced dehydration on competitive running performance. Med. Sci. Sports Exerc. 17:456–461. 4033401 10.1249/00005768-198508000-00009

[phy213315-bib-0002] Arnaoutis, G. , S. A. Kavouras , I. Chrstaki , and L. S. Sidossis . 2012 Water ingestion improves performance compared with mouth rinse in dehydrated subjects. Med. Sci. Sports Exerc. 44:175–179. 21685819 10.1249/MSS.0b013e3182285776

[phy213315-bib-0003] Bardis, C. N. , S. A. Kavouras , G. Arnaoutis , D. B. Panagiotakos , and L. S. Sidossis . 2013a Mild dehydration and cycling performance during 5‐kilometer hill climbing. J. Athl. Train. 48:741–747. 23952038 10.4085/1062-6050-48.5.01 PMC3867084

[phy213315-bib-0004] Bardis, C. N. , S. A. Kavouras , L. Kosti , M. Markousi , and L. S. Sidossis . 2013b Mild hypohydration decreases cycling performance in the heat. Med. Sci. Sports Exerc. 45:1782–1793. 23470313 10.1249/MSS.0b013e31828e1e77

[phy213315-bib-0005] Below, P. R. , R. Mora‐Rodriguez , J. Gonzalez‐Alonso , and E. F. Coyle . 1995 Fluid and carbohydrate ingestion independently improve performance during 1 h of intense exercise. Med. Sci. Sports Exerc. 27:200–210. 7723643

[phy213315-bib-0006] Berkulo, M. A. , S. Bol , K. Levels , R. P. Lamberts , H. A. Daanen , and T. D. Noakes . 2016 Ad‐libitum drinking and performance during a 40‐km cycling time trial in the heat. Eur. J. Sport Sci. 16:213–220. 25675355 10.1080/17461391.2015.1009495

[phy213315-bib-0007] Borg, G. A. 1982 Psychophysical bases of perceived exertion. Med. Sci. Sports Exerc. 14:377–381. 7154893

[phy213315-bib-0008] Casa, D. J. , Ganio, M. S. , Lopez, R. M. , McDermott, B. P. , Armstrong, L. E. , and Maresh, C. M . 2008 Intravenous versus oral rehydration: physiological, Performance, and Legal considerations. Curr. Sports Med. Rep. 4:S41–S49.

[phy213315-bib-0009] Casa, D. J. , R. L. Stearns , R. M. Lopez , M. S. Ganio , B. P. McDermott , S. Walker Yeargin , et al. 2010 Influence of hydration on physiological function and performance during trail running in the heat. J. Athl. Train. 45:147–156. 20210618 10.4085/1062-6050-45.2.147 PMC2838466

[phy213315-bib-0010] Castellani, J. W. , S. R. Muza , S. N. Cheuvront , I. V. Sils , C. S. Fulco , R. W. Kenefick , et al. 2010 Effect of hypohydration and altitude exposure on aerobic exercise performance and acute mountain sickness. J. Appl. Physiol. 109:1792–1800. 20864559 10.1152/japplphysiol.00517.2010

[phy213315-bib-0011] Cheung, S. S. , G. W. McGarr , M. M. Mallette , P. J. Wallace , C. L. Watson , I. M. Kim , et al. 2015 Separate and combined effects of dehydration and thirst sensation on exercise performance in the heat. Scand. J. Med. Sci. Sports 25(Suppl 1):104–111. 10.1111/sms.12343 25943661

[phy213315-bib-0012] Cheuvront, S. N. , and R. W. Kenefick . 2014 Dehydration: physiology, assessment, and performance effects. Compr. Physiol. 4:257–285. 24692140 10.1002/cphy.c130017

[phy213315-bib-0013] Cheuvront, S. N. , R. Carter III , J. W. Castellani , and M. N. Sawka . 2005 Hypohydration impairs endurance exercise performance in temperate but not cold air. J. Appl. Physiol. 99:1972–1976. 16024524 10.1152/japplphysiol.00329.2005

[phy213315-bib-0014] Cotter, J. D. , S. N. Thornton , J. K. Lee , and P. B. Laursen . 2014 Are we being drowned in hydration advice? Thirsty for more? Extrem. Physiol. Med. 3:18. 25356197 10.1186/2046-7648-3-18 PMC4212586

[phy213315-bib-0015] Davis, B. A. , L. K. Thigpen , J. H. Hornsby , J. M. Green , T. E. Coates , and E. K. O'Neal . 2014 Hydration kinetics and 10‐km outdoor running performance following 75% versus 150% between bout fluid replacement. Eur. J. Sport Sci. 14:703–710. 24697790 10.1080/17461391.2014.894578

[phy213315-bib-0016] Dill, D. B. , and D. L. Costill . 1974 Calculation of percentage changes in volumes of blood, plasma, and red cells in dehydration. J. Appl. Physiol. 37:247–248. 4850854 10.1152/jappl.1974.37.2.247

[phy213315-bib-0017] Dugas, J. P. , U. Oosthuizen , R. Tucker , and T. D. Noakes . 2009 Rates of fluid ingestion alter pacing but not thermoregulatory responses during prolonged exercise in hot and humid conditions with appropriate convective cooling. Eur. J. Appl. Physiol. 105:69–80. 18853180 10.1007/s00421-008-0876-6

[phy213315-bib-0018] Ebert, T. R. , D. T. Martin , N. Bullock , I. Mujika , M. J. Quod , L. A. Farthing , et al. 2007 Influence of hydration status on thermoregulation and cycling hill climbing. Med. Sci. Sports Exerc. 39:323–329. 17277597 10.1249/01.mss.0000247000.86847.de

[phy213315-bib-0019] Figaro, M. K. , and G. W. Mack . 1997 Regulation of fluid intake in dehydrated humans: role of oropharyngeal stimulation. Am. J. Physiol. 272:R1740–1746. 9227585 10.1152/ajpregu.1997.272.6.R1740

[phy213315-bib-0020] Fleming, J. , and L. J. James . 2014 Repeated familiarisation with hypohydration attenuates the performance decrement caused by hypohydration during treadmill running. Appl. Physiol. Nutr. Metab. 39:124–129. 24476466 10.1139/apnm-2013-0044

[phy213315-bib-0021] Frayn, K. N. 1983 Calculation of substrate oxidation rates in vivo from gaseous exchange. J. Appl. Physiol. 55:628–634. 6618956 10.1152/jappl.1983.55.2.628

[phy213315-bib-0022] Gonzalez‐Alonso, J. , J. Calbet , and B. Nielsen . 1998 Muscle blood flow is reduced with dehydration during prolonged exercise in humans. J. Physiol. 513:895–905. 9824726 10.1111/j.1469-7793.1998.895ba.x PMC2231307

[phy213315-bib-0023] Goulet, E. D. 2011 Effect of exercise‐induced dehydration on time‐trial exercise performance: a meta‐analysis. Br. J. Sports Med. 45:1149–1156. 21454440 10.1136/bjsm.2010.077966

[phy213315-bib-0024] Kenefick, R. W. , S. N. Cheuvront , L. J. Palombo , B. R. Ely , and M. N. Sawka . 2010 Skin temperature modifies the impact of hypohydration on aerobic performance. J. Appl. Physiol. 109:79–86. 20378704 10.1152/japplphysiol.00135.2010

[phy213315-bib-0025] Logan‐Sprenger, H. M. , G. J. Heigenhauser , G. L. Jones , and L. L. Spriet . 2015 The effect of dehydration on muscle metabolism and time trial performance during prolonged cycling in males. Physiol. Rep. 3:e12483. 26296770 10.14814/phy2.12483 PMC4562569

[phy213315-bib-0026] McClung, M. , and D. Collins . 2007 “Because I know it will!”: placebo effects of an ergogenic aid on athletic performance. J. Sport. Exerc. Psychol. 29:382–394. 17876973 10.1123/jsep.29.3.382

[phy213315-bib-0027] McConell, G. K. , C. M. Burge , S. L. Skinner , and M. Hargreaves . 1997 Influence of ingested fluid volume on physiological responses during prolonged exercise. Acta Physiol. Scand. 160:149–156. 9208041 10.1046/j.1365-201X.1997.00139.x

[phy213315-bib-0028] McConell, G. K. , T. J. Stephens , and B. J. Canny . 1999 Fluid ingestion does not influence intense 1‐h exercise performance in a mild environment. Med. Sci. Sports Exerc. 31:386–392. 10188742 10.1097/00005768-199903000-00006

[phy213315-bib-0029] Merry, T. L. , P. N. Ainslie , and J. D. Cotter . 2010 Effects of aerobic fitness on hypohydration‐induced physiological strain and exercise impairment. Acta Physiol. 198:179–190. 10.1111/j.1748-1716.2009.02051.x 19807723

[phy213315-bib-0030] Montain, S. J. , and E. F. Coyle . 1999 Influence of graded dehydration on hyperthermia and cardiovascular drift during exercise. J. Appl. Physiol. 73:1340–1350. 10.1152/jappl.1992.73.4.1340 1447078

[phy213315-bib-0031] Saunders, A. G. , J. P. Dugas , R. Tucker , M. I. Lambert , and T. D. Noakes . 2005 The effects of different air velocities on heat storage and body temperature in humans in a hot, humid environment. Acta Physiol. Scand. 183:377–390. 10.1111/j.1365-201X.2004.01400.x 15743384

[phy213315-bib-0032] Sawka, M. N. , A. J. Young , R. P. Francesconi , S. R. Muza , and K. B. Pandolf . 1985 Thermoregulatory and blood responses during exercise at graded hypohydration levels. J. Appl. Physiol. 59:1394–1401. 4066570 10.1152/jappl.1985.59.5.1394

[phy213315-bib-0033] Sawka, M. N. , L. M. Burke , E. R. Eichner , R. J. Maughan , S. J. Montain , and N. S. Stachenfeld . 2007 American College of Sports Medicine position stand. Exercise and fluid replacement. Med. Sci. Sports Exerc. 39:377–390. 17277604 10.1249/mss.0b013e31802ca597

[phy213315-bib-0034] Sawka, M. N. , S. N. Cheuvront , and R. W. Kenefick . 2015 Hypohydration and human performance: impact of environment and physiological mechanisms. Sports Med. 45(Suppl 1):51–60. 10.1007/s40279-015-0395-7 PMC4672008 26553489

[phy213315-bib-0035] Stearns, R. L. , D. J. Casa , R. M. Lopez , B. P. McDermott , M. S. Ganio , N. R. Decher , et al. 2009 Influence of hydration status on pacing during trail running in the heat. J Strength Cond Res 23:2533–4251. 19675477 10.1519/JSC.0b013e3181b73c3f

[phy213315-bib-0036] Stewart, C. J. , D. G. Whyte , J. Cannon , J. Wickham , and F. E. Marino . 2014 Exercise‐induced dehydration does not alter time trial or neuromuscular performance. Int. J. Sports Med. 35:725–730. 24577860 10.1055/s-0033-1364022

[phy213315-bib-0037] Trangmar, S. J. , S. T. Chiesa , C. G. Stock , K. K. Kalsi , N. H. Secher , and J. González‐Alonso . 2014 Dehydration affects cerebral blood flow but not its metabolic rate for oxygen during maximal exercise in trained humans. J. Physiol. 592:3143–3160. 24835170 10.1113/jphysiol.2014.272104 PMC4214665

[phy213315-bib-0038] Wall, B. A. , G. Watson , J. J. Peiffer , C. R. Abbiss , R. Siegel , and P. B. Laursen . 2015 Current hydration guidelines are erroneous: dehydration does not impair exercise performance in the heat. Br. J. Sports Med. 49:1077–1083. 24055782 10.1136/bjsports-2013-092417

[phy213315-bib-0039] Walsh, R. M. , T. D. Noakes , J. A. Hawley , and S. C. Dennis . 1994 Impaired high‐intensity cycling performance time at low levels of dehydration. Int. J. Sports Med. 15:392–398. 8002117 10.1055/s-2007-1021076

